# ‘Shall We Send a Panda?’ A Practical Guide to Engaging Schools in Research: Learning from Large-Scale Mental Health Intervention Trials

**DOI:** 10.3390/ijerph19063367

**Published:** 2022-03-12

**Authors:** Anna Moore, Emma Ashworth, Carla Mason, Joao Santos, Rosie Mansfield, Emily Stapley, Jessica Deighton, Neil Humphrey, Nick Tait, Daniel Hayes

**Affiliations:** 1 Evidence Based Practice Unit (EBPU), Anna Freud National Centre for Children and Families and University College London (UCL), 4-8 Rodney Street, London N1 9JH, UK; emily.stapley@annafreud.org (E.S.); jessica.deighton@annafreud.org (J.D.); daniel.hayes@annafreud.org (D.H.); 2 School of Psychology, Liverpool John Moores University, Liverpool L3 5UX, UK; e.l.ashworth@ljmu.ac.uk; 3 Manchester Institute of Education, The University of Manchester, Manchester M13 9PL, UK; carla.mason@manchester.ac.uk (C.M.); joao.santos@manchester.ac.uk (J.S.); neil.humphrey@manchester.ac.uk (N.H.); 4 Centre for Longitudinal Studies, University College London, London WC1E 6BT, UK; r.mansfield@ucl.ac.uk; 5 Anna Freud National Centre for Children and Families (AFNCCF), London N1 9JH, UK; nick.tait@annafreud.org

**Keywords:** school based, trials, barriers, facilitators, engagement, research

## Abstract

The substantial time that children and young people spend in schools makes them important sites to trial and embed prevention and early intervention programmes. However, schools are complex settings, and it can be difficult to maintain school engagement in research trials; many projects experience high levels of attrition. This commentary presents learning from two large-scale, mixed-methods mental health intervention trials in English schools. The paper explores the barriers and challenges to engaging schools in promotion or early intervention research and offers detailed recommendations for other researchers.

## 1. Introduction

### 1.1. Schools as a Site for Health and Prevention Research

The substantial time that pupils spend in schools makes them one of the most convenient and efficient places to obtain data from large, representative, and universal samples of young people [1,2,3]. With an increasing focus in recent years on schools’ role in promoting health, many research trials are taking place to determine the effectiveness of health and wellbeing interventions in schools and to explore factors affecting implementation [4]. Participating in such research can be advantageous to schools, often providing access to staff training and, in some cases, even improving academic performance [5]. However, teachers in English schools already work longer hours than many of their counterparts in other developed countries, and school leaders are under substantial financial pressure [6,7]. Engaging schools in research presents many challenges and projects often experience high levels of attrition, which, in turn, introduces a source of bias [8,9]; despite multiple benefits for researchers, policymakers, and schools themselves, schools are complex sites for research. 

### 1.2. The Complexity of Schools

While schools prove very valuable for conducting research with children and young people, the school setting presents unique challenges. Schools are best recognised as social complex adaptive systems (SCASs) that, ‘comprise a population of diverse rules-based agents, located in multi-level and interconnected systems in a network shape’ [10] (p. 1468). These SCASs are made up of multiple nested systems with distributed control; schools consist of individual ‘agents’ (e.g., staff members, parents, students) and subsystems (e.g., subject departments, year groups) that have varying degrees of interaction [10]. Schools themselves are then nested in a larger, densely interconnected system of education, influenced by higher-level factors such as government and local authority policies [11]. Viewing schools as SCASs allows for a better understanding of their processes, rules, and behaviours [10]. This helps to inform strategies that can be put in place to overcome common hurdles to school-based research. 

### 1.3. The Importance of Staff Engagement

Of key relevance to school-based research is the role of the staff ‘agents’ in the SCAS. While they typically act in ways that are based on a combination of their knowledge, experience, feedback from the environment, local values, and both formal and informal (e.g., school ethos) system rules, they also have a relatively high degree of autonomy and are diverse in terms of attitudes, opinions, and resources [12,13]. As a consequence, access to schools can vary significantly based on the interests, priorities, and willingness of the staff member(s) involved in the recruitment and ongoing administration of the research [14]. Schools in England also often form part of large multi-academy trusts, where trust leadership and budget allocation have a sizeable impact on individual schools [15]. Engagement and priority setting by these higher-level bodies can also affect school participation in research [16]. 

In terms of the wider school environment, factors such as the systems for sharing information between staff members, the school ethos, policies, hierarchy, and organisational structure can all influence recruitment and retention in research studies. Schools’ goals and the priorities of members of the senior leadership team and governing body will be paramount when a school is deciding whether or not to participate in a research project [14]. However, even with engagement from members of the senior leadership team, if the school has poor information sharing methods that result in vital information regarding the research not being successfully disseminated to the key staff members involved, the project will stall and ultimately fail [10].

### 1.4. Competing Priorities

More broadly, time available in schools is a key barrier to research in this setting [16]. Schools face multiple competing priorities and external pressures, particularly at certain times of the year (e.g., examination periods). They are continually measured on their ability to meet specific academic outcomes and are required to cover an extensive curriculum [6]. Finding time for a staff member to complete the necessary administration tasks associated with research studies (e.g., consent forms, arranging resources), as well as space in school timetables for the research to be conducted, is a challenge facing all schools involved in research activity [1,14,17]. The success of a research study in a school is likely to be dependent on a complex interplay of the factors noted above. It is clear that methods to mitigate these are needed to lessen the burden on schools, reduce the significant attrition rates common to this setting, and ensure effective school-based research. 

### 1.5. The Education for Wellbeing Programme

The Education for Wellbeing (EfW) programme, commissioned by the Department for Education (DfE), is England’s largest research trial of school-based mental health interventions [18,19]. With the rising rates of child mental health difficulties in recent years [20], schools are increasingly being recognised as key sites to embed prevention and early intervention programmes [21]. Recent policy in England increases schools’ responsibility to support pupils’ mental health and introduces compulsory mental health education [22,23]. In light of this, the trial is being conducted to understand ‘what works’ in the English school context. The EfW programme is working with approximately 370 mainstream primary and secondary schools to evaluate five mental health and wellbeing interventions. The programme is split into two randomised controlled trials: AWARE, with year 9 pupils (aged 13–14 years), and INSPIRE, with years 4, 5, 7, and 8 (aged 8–13 years). Of the five interventions, three aim to reduce emotional difficulties, and two aim to increase help-seeking intentions. Prior to the main trials, a feasibility study was conducted (2017–2018) with 20 schools. Full details on the research design and methodology of the randomised controlled trials are available in the AWARE and INSPIRE protocol papers [18,19].

In addition to exploring effectiveness, the research also involves an economic strand and an implementation and process monitoring strand. These strands involve multiple surveys for staff and pupils across baseline and follow-ups, as well as obtaining parental and pupil consent. The research also requires organising the delivery of interventions (e.g., timetabling allocations, provision of resources, and releasing staff for training). As well as the strands mentioned above, a subset of schools in the first year of the programme also facilitated qualitative case study visits. This involved additional consent procedures and arranging qualitative interviews and focus groups with staff and pupils. Ethical approval for the EfW trials was granted by the University College London (UCL) Research Ethics Committee (6735/009 and 6735/014). 

In this paper, we present our learning from engaging with schools through the EfW research programme and offer recommendations for other researchers in similar fields looking to work with schools. Our aim is to share our learning from this large and complex project to help others avoid some of the pitfalls we faced and draw on some of the techniques we found useful. While some of the approaches to research discussed here will be specific to the English context, we believe the learning from this project illustrates many of the key challenges that arise when conducting mental health research in school settings.

We provide a summary of our findings in Table 1, split into the following overarching categories: (1) general challenges, (2) staff engagement, (3) communicating with school staff, (4) quantitative research, and (5) qualitative research. Each of these categories is then discussed in further detail below in relation to challenges and solutions within the context of the EfW programme. 

## 2. Key Learning

### 2.1. General Challenges

#### 2.1.1. Recruitment

The EfW programme benefitted from being funded by the DfE; colleagues at DfE had a wider reach than our research unit and were able to advertise the programme on several different platforms. We also found that, for some members of school staff, the fact that the programme was funded by the government seemed to legitimise the research. 

However, for those starting a research project in schools without government funding, we recommend trying to directly contact the school staff working in relevant roles (e.g., Mental Health Lead, Special Educational Needs and Disabilities Coordinator (SENDCo)). We found this to be more successful than sending advertisements to generic school email addresses. As discussed above, schools are tied into wider networks and are influenced by local authorities. Contact with local authorities and multi-academy trusts can prove helpful in promoting and recommending the research to wider groups of schools. 

#### 2.1.2. School Attrition

Despite good engagement from school staff at the beginning of the research, we found it important to plan for the attrition of schools throughout the project. Across two years of the EfW programme, 40% of eligible schools that expressed interest (the first stage of recruitment) did not proceed to complete the Memorandum of Understanding and commit to the research project (the second stage of recruitment). Of those that signed the Memorandum of Understanding and started the research, 20% of schools did not complete the baseline data collection. 

There were a number of reasons for this school-level attrition, even in schools with high levels of engagement early on in the project. Some of the reasons provided for school drop-out included changes in school leadership, difficulties timetabling the interventions, concerns about sharing pupil data, lack of capacity to complete surveys, and difficulties releasing staff for training. To combat attrition, we recommend factoring the over-recruitment of schools into funding applications and study design. It is also important that a senior member of staff (and perhaps a school governor) signs a Memorandum of Understanding so that they are aware of the project requirements. We recommend creating a waiting list for interested schools—if a school fails to sign the necessary documentation by a certain date, you can offer their place to another school. 

In the EfW programme, we were also able to offer schools a payment of GBP 1000 on completion of the research, along with a feedback report, a signed letter of thanks from the DfE, and a participation certificate. We also provided schools with a feedback report capturing aggregate data on pupil wellbeing; schools reported that this was useful in planning for future provision. These incentives helped reduce attrition, particularly in the final stages of the research. If school staff had moved on or stopped responding to research communications, we were able to contact senior members of school staff and offer these benefits in return for the completion of the final surveys. Though GBP 1000 is a small sum in relation to their overall budget, schools reported that this money helped to pay for cover for staff attending training, printing costs, and also further programmes of work around mental health and wellbeing at the end of the research.

#### 2.1.3. Understanding the Specific Challenges of Your Research

Every research project will face a unique set of challenges when engaging and working with schools. As part of the EfW programme, we conducted a feasibility study with 20 schools to trial all aspects of our research, including the survey measures, facilitating pupil surveys, acceptability of the five different interventions, interview schedules for qualitative visits, and both our internal and external communication systems. The feasibility study also helped shape our understanding of school dropouts and our timelines for the recruitment of schools. We recommend building a feasibility or pilot study into funding applications for school-based research to allow time for informed planning and to ensure issues (with interventions, timelines, research personnel, communications) have been addressed before the rollout of the main trial.

### 2.2. Staff Engagement

School staff members have complex roles, and it can be difficult to secure engagement from staff, particularly in secondary schools, where significant hierarchies exist. We found difficulties both when a junior member of staff was trying to proceed without senior involvement, and when a senior member of staff committed to the research without buy-in from the classroom teachers who would be required to do the work. School staff in all roles are faced with many competing priorities and will, in the majority of cases, see the research processes as additional work. In our experience, staff members were not relieved of other responsibilities in order to participate in the EfW programme but were instead fitting the research project into an already busy working week. 

We found school staff to be more willing to engage in research if they understood its purpose and could see the benefits of the work they are putting in for their pupils, their school, their teaching practice, as well as for pupils across the country. It is important to reiterate the goals of the research throughout the project and highlight the significance of their contribution at every opportunity. In email communication, phone calls, and newsletters to schools, we consistently expressed our gratitude to schools and emphasised the important contribution they are making to an accruing evidence base.

Members of staff involved in the project will approach the research with differing levels of experience and may not have participated in research previously. Clear communication about research aims, methods, and potential impact will help with research literacy and, as a consequence, improve staff motivation. Improved research literacy among staff can also lead to this filtering down to pupils. If there is an opportunity to meet the staff (e.g., at an intervention training session), it is beneficial for a member of the research team to provide an overview of the evaluation and answer any questions; meeting researchers face-to-face can really help to build positive relationships with school staff. 

Members of school staff are very unlikely to engage in work with external researchers if they feel that the research team does not understand the school setting, or if they feel that research tasks or timelines are unrealistic. It is important to demonstrate in all communication with school staff that each stage of the research process has been carefully considered. This can involve researchers checking the dates of all school holidays and planning survey deadlines accordingly or ensuring that particularly busy times of the school year (e.g., exam periods) do not coincide with key research milestones. Whilst much of this work can be completed in advance, it is important to be flexible and understanding if school staff present issues that had not been predicted. Where possible, it is valuable to hire researchers with substantial experience of working in and with schools; this can help with the perceived credibility and understanding of the research team.

If schools are falling behind on research steps, personalised communication is much more effective than generic emails and helps staff to build relationships with the research team. It is crucial to sign off emails personally and ensure that staff members know who to come to with any issues.

### 2.3. Communicating with School Staff 

#### 2.3.1. Emails

**Figure 1:**
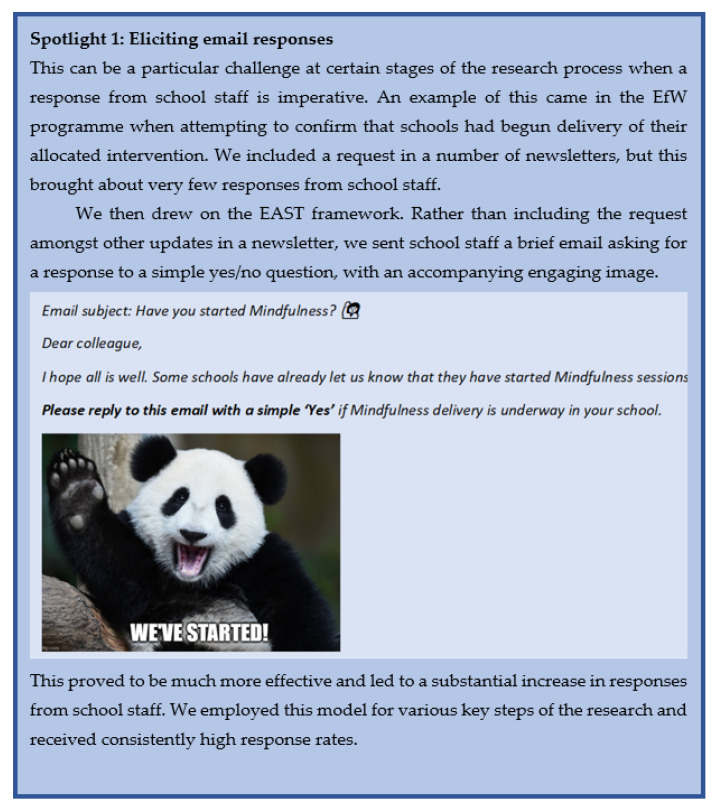
Spotlight on Eliciting Email Responses. Panda image: V-yan (Photographer) A playful happy panda in China (digital image) retrieved from https://www.shutterstock.com/image-photo/playful-happy-panda-china-1390386575 (accessed on 10 January 2022).

School staff are incredibly busy, and the nature of their working day leaves very little time for extra administration. They receive numerous emails throughout the school day but are rarely able to sit down and respond. We found that sending regular newsletters and brief emails to schools (File S2) helped them to keep on track with the research and understand what was required of them at any given time. If school staff had missed a previous newsletter, there was no need to sift through their inbox to find information as it would pop up again the following week. At times we also made use of the EAST technique (Figure 1) to make the behaviour easy, attractive, social, and timely [24]. 

It is important to structure emails carefully to include the essential points first, and it is helpful to limit key steps or information to three per email. If there are documents to attach, numbering attachments and making sure they correspond to steps in the body of the email makes it much easier to follow (e.g., print DOC 1 for pupils, send DOC 2 to parents/carers). Where possible, it is best to send information directly to specific staff members (e.g., intervention deliverers or staff facilitating the surveys) as information sent to one school contact may not always be passed on in a timely fashion. The volume of emails and the pressures on school staff may lead to some staff members missing deadlines, such as the deadline for facilitating pupil surveys. During a survey period, it is helpful to include a countdown in the weekly newsletter (File S2) and provide regular updates on each school’s completion rate. 

As a result of school holidays, staff can also be out of contact for significant periods of the year (in England, this usually equates to around 13 weeks in total). Holiday periods will vary between different types of schools (e.g., primary, secondary, independent, faith schools) and different geographical locations, so researchers should invest time in gathering this information on participating schools. Whilst some members of staff may check their emails occasionally during holidays, the research team should not rely on this. In addition to avoiding busy exam periods, all research timelines and communication should be planned carefully around holidays. 

#### 2.3.2. Phone Calls

There may be only one timeslot in a given week where a teacher will be able to speak on the phone. Phone calls work better when an appointment is made in advance with school staff, and any document or information is sent over beforehand. Having multiple contacts increases the possibility of speaking to someone who is informed about the research. If it is not possible to reach a school contact, and there is an urgent request, the next best action is to leave a message with reception staff, emphasising key tasks and the benefits of taking part.

#### 2.3.3. Contacting the Right People

Successful school-based research often requires the involvement of multiple staff members; engagement and commitment from the right people in a school will make or break the project. Having contact details of more than one member of school staff has proved invaluable throughout our research. In the EfW programme, we collected details of one senior member of staff (also responsible for overall sign-off), a key contact, and a second contact in the initial expression of interest form. Regular communication was sent to the key contact and second contact (e.g., weekly newsletters), and these two members of staff were also occasionally allocated distinct tasks. We generally avoided contacting the senior members of staff with the day-to-day running of the project, but we found it very useful to include senior leadership when we had not heard from the other contacts or when a school was close to missing an important deadline. Due to the size and complexity of secondary schools, we also recommend an additional contact for these settings. For example, if you are evaluating an intervention delivered by class teachers, it would be beneficial to have details of a member of staff with oversight who would be able to disseminate important information (e.g., a Head of Year in a UK school setting).

#### 2.3.4. Staff Turnover

Whilst we had details of three contacts for each school, we underestimated the turnover of school staff, especially at the end of the academic year. This resulted in a number of delays when organising follow-up data collection, and we recommend checking regularly that the contact details are up to date. At the end of the school term, it is a good idea to remind staff to notify the research team if they are leaving and to provide details for the person who will be assuming their role.

If several emails have remained unanswered, or there is a bounce-back notification, do not hesitate to phone the school and speak to reception staff to resolve this issue. School email addresses may also change as a result of renaming or rebranding (e.g., when schools may merge with others to form an academy trust in the UK), so it is important to follow up. Unfortunately, there were instances in which we wrongly assumed that schools had been following the research steps, only to discover months later that none of our newsletters had been received. 

#### 2.3.5. Large Research Projects

If the research involves a large number of schools, there will often be particularly busy periods during which all schools contact the team and require assistance at the same time. To avoid delays to project timelines, it is necessary to respond to any queries as soon as possible. For example, a deputy headteacher may only have time on Monday afternoons to work on the project, so if a researcher does not respond until Tuesday, the progress of that school may be set back by a week. It may also be worth investing in communications software or a marketing platform (e.g., Mailchimp) to store contact details and create newsletters, ensuring that a member of the team has experience with communications.

Researchers should carefully consider allocating clear roles within their team with respect to school communication (e.g., some providing technical survey support, others dealing with general queries) and may consider hiring additional administration support for short periods of the project to cope with the volume of emails and phone calls. It is crucial that all members of the research team have access to the same information and that this is kept up to date (make use of software such as Microsoft Teams or Slack to ensure ‘live’ editing of spreadsheets). Creating a rota for the research team to oversee inboxes and spreadsheets can be useful in particularly busy periods. It is also helpful to create standardised communications and answers to frequently asked questions and store them centrally so all members of the research team can respond without replicating work. 

### 2.4. Quantitative Research

**Figure 2:**
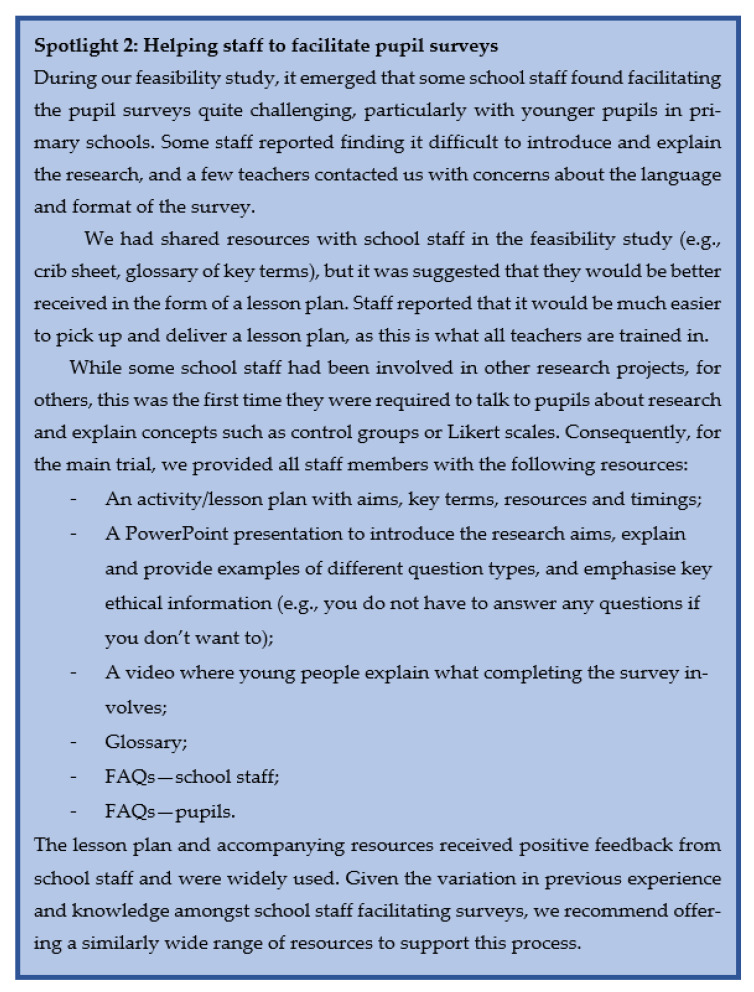
Spotlight on helping staff to facilitate pupil surveys.

While online surveys are a very efficient way to collect data from a large sample, this can be complicated in schools, where every minute of the day is already timetabled and staff members do not often have easy access to laptops or computer rooms to facilitate the survey. It is important to provide schools with advance notice for survey periods, send regular reminders when the survey is approaching, and provide as much help as possible for staff facilitating the pupil surveys (Figure 2). This will allow staff to book computer rooms and prepare for their pupils to complete the survey. Providing resources to help with research literacy is also important to help staff convey the research aims and wider context to pupils [25].

A key part of this preparation is asking staff to test their IT systems in advance of the planned session with pupils; there are often issues with school IT systems, including extensive safety and privacy measures, that prohibit staff from accessing certain links or programmes. Where possible, it is best to communicate directly with the school’s IT department and resolve any technical issues directly with them. It may be a good idea to collect the contact details of a member of the school’s IT department at the beginning of the research project, especially when working with large secondary schools.

Despite the best efforts of school staff, there will be times when they are unable to meet project deadlines for surveys. It is important when planning school-based research to allow for this and to offer schools a grace period in case of unexpected difficulties.

### 2.5. Qualitative Research

Working with pupils below the age of 16 requires parental consent, and these ethical processes involve additional administration by school staff. We learned that it is crucial to allow enough time for these processes (at least 2–3 weeks before the visit) and to provide the school with a timeline outlining clear steps to obtain consent. It is helpful to send a reminder one week before the visit to check that the necessary processes are underway and that staff members have organised focus groups and booked private rooms for qualitative interviews or focus groups.

During a case study school visit, the staff members who have organised the day will likely be teaching and unable to assist researchers. A checklist of everything researchers need for the day is useful, including personal documentation (e.g., DBS), all research documentation printed out in advance (with spares), and details of the safeguarding lead; the research team must be able to be self-sufficient in the school.

Despite sharing clear information in advance, stretched school staff may not always carry out research processes in the correct way. Where possible, it helps to align with the school’s usual consent processes (e.g., sending letters out to parents/carers or gaining consent by email), and it is useful to provide schools with more than one option. Before the visit, it is important to follow up with schools and confirm with staff members that they have followed the given processes to obtain parental consent. 

## 3. Conclusions

Member of school staff spend long hours working with pupils and their parents/carers, delivering an extensive curriculum under pressure to produce outcomes on many levels. In this environment, participating in research can be the least important commitment in a large and shifting mosaic of priorities for school staff. The framing of schools as complex adaptive systems is useful in helping us to understand factors affecting their engagement in research [10]. This allows us to shape research design and processes to facilitate engagement at all levels (e.g., working with staff in a variety of roles, gaining the support of local authorities, providing resources for staff with a wide range of research experience). 

It is in the interests of all involved to form collaborative relationships and facilitate school engagement in research; schools can benefit from participating in research whilst making an important contribution to shape evidence-based practice and policy [21]. While the challenges and solutions explored in this trial in England may be similar to those in other countries, it is important to acknowledge the considerable variation across the globe in educational approaches, opportunities, and capacity. School resources, priorities, and systems of governance vary widely across countries, and this will create diverse environments for conducting research in schools [26,27].

A limitation of this paper is the specific English context, where all schools have access to computers and laptops for pupils, and all teachers use email communication every day in their work. We acknowledge that researchers working in low- and middle-income countries will not necessarily be using online surveys and emails; thus, the sections here on quantitative research and emailing school staff may be less relevant. The structure of the education system in England, where all state schools are under the governance of regional ‘Local Authorities’, which, in turn, are answerable to the English government’s Department for Education, also benefitted our communications. However, the challenges around attrition, staff engagement in research, and the approach to communication are generalisable to all settings. Our findings from the EfW programme highlight the need for careful planning and consideration from the conception of a research project. Research processes must be designed specifically for the school context and practical implications considered before the recruitment of schools. Communication, both directly with school staff and amongst the research team, is key to a successful project and time must be given to ensuring information is clear and consistent. While every effort must be made to prepare for the complexity outlined here, each research project will face its own challenges, and the research team will also need to be accommodating and flexible in response to schools’ needs.

We hope the experiences and recommendations shared here will help researchers foster positive relationships with school staff and lead to successful engagement in future research projects.

## Figures and Tables

**Table 1:** Key challenges and solutions in the Education for Wellbeing programme.

Category	Challenge	Solutions
1. General challenges	- Accessing the right staff member for recruitment; - Significant school-level attrition; - Unique challenges for each research project.	- Contact staff responsible for health/wellbeing/inclusion; - Over-recruit schools; - Conduct a feasibility study; - Provide schools with incentives (e.g., payment, certificates).
2. Staff engagement	- Research requires engagement from staff members in different roles; - Staff have complex roles and limited capacity.	- Make aims of research clear and outline the importance of their contribution (links to pupil engagement); - Demonstrate understanding of the school context and careful consideration of their time; - Use personalised communication where possible.
3. Communicating with school staff	- Staff have very little time for research administration; - Staff receive many emails and may miss important information; - Contacting staff by phone is difficult; - Staff turnover; - Large research projects require additional support for school communications.	- Break the research down into clear phases and steps (File S1); - Send regular newsletters outlining key tasks for staff (File S2); - Use the EAST technique to elicit responses (Figure 1); - Book appointments for phone calls; - Work with multiple members of staff and regularly check contact details; - Plan carefully for busy periods when working with large numbers of schools and make use of marketing software.
4. Quantitative research	- Accessing laptops/computer rooms to facilitate surveys; - School IT systems; - Varied research experience amongst school staff.	- Provide advance notice for survey periods and remind staff to book computer rooms; - Liaise directly with school IT departments where possible; - Provide a range of resources to help with research literacy (Figure 2).
5. Qualitative research	- Additional parental consent processes; - Staff capacity during case study visits.	- Provide schools with clear timelines for case study visits; - Conduct multiple check-ins with school staff before the visit.

## Data Availability

Not applicable.

## Supplementary Materials

The following are available online at https://www.mdpi.com/article/10.3390/ijerph19063367/s1. File S1: Example research phases and dates, File S2: Example school newsletter.
