# Supplementary material for: ‘Shall We Send a Panda?’ A Practical Guide to Engaging Schools in Research: Learning from Large-Scale Mental Health Intervention Trials
Source: Int J Environ Res Public Health. 2022 Mar 12;19(6):3367. doi: 10.3390/ijerph19063367 (PMC8950538; doi:10.3390/ijerph19063367)
Supplement: Supplementary file 1 [file ijerph-19-03367-s001.zip › ijerph-1580183-supplementary.pdf]

Supplemental Materials

File S1. Example Research Phases

Phase	Task	Description	Date
**Phase 1 – Preparation:**	**1. Memorandum of Understanding**	The Memorandum of Understanding needs to be signed and returned by email to the Education for Wellbeing team.	May 18
	**2. Sign data sharing agreement**	The data sharing agreement needs to be signed and returned by email to the Education for Wellbeing team.	May 18
	**3. Hold training dates for teachers**	As schools will only be notified of their group after the Baseline Data Collection phase, we will need all schools to hold the training dates provided.	May 18
	**4. Share lists of delivery groups**	The Key Contact will need to share lists of the pupils who will be taking part in the programme with our evaluation team.	May 18
	**5. Send information to parents/carers**	Schools will need to send out information sheets drafted by the evaluation team – this will include a broad outline of the project and opt-out forms for parents to return directly to us.	June 18
**Phase 2 – Baseline Data Collection:**	**1. Complete the Existing Provision Survey**	The Key Contact will fill out a questionnaire about the mental health and wellbeing provision that is already taking place in your school.	June 18
	**2. Complete Teacher Surveys and Pupil Surveys (Time 1)**	Teachers and pupils will need to complete online questionnaires. A computer room will need to be booked for pupils.	Sept 18
**Phase 3 – Delivery training:**	**1. Receive news of allocation**	After October half-term we will contact schools to inform them of the group they have been allocated to. Schools will also be made aware if they are a case study school (see Phase 4 below). A link to book onto relevant training dates (depending on your allocation) will be sent around to schools.	Nov 18
	**2. Teachers attend training**	Schools allocated to one of the Mindfulness, Relaxation or Strategies for Safety & Wellbeing groups will release teachers for 0.5 day training.	Nov – Dec 18
**Phase 4 – Delivery:**	**1. Intervention delivery**	Schools running these interventions will deliver these programmes, while Usual Practice schools will continue as normal.	Jan-March 19
	**2. Case Study School visits**	Selected schools will take part in interviews, focus groups and observations with a small number of staff and pupils. Schools will be made aware of whether or not this is required in November 18.	Jan-March 19
**Phase 5 – Initial Follow-Up Data Collection:**	1. **Complete Implementation Surveys (Time 1)**	This will only be completed by teachers in schools delivering the interventions (not Usual Practice Schools)	Feb-March 19
	2. **Complete Finance Survey**	A member of the finance team at your schools will need to share some financial information so we can work out how much the interventions cost to deliver.	May 19
	3. **Complete Teacher Surveys (Time 2) and Pupil Surveys (Time 2)**	Follow up questionnaires will need to be completed by pupils and teachers. A computer room will need to be booked for pupils.	Jun 19
**Phase 6 – Final Follow-Up Data Collection:**	1. **Complete Teacher Surveys (Time 3)**	Follow up questionnaires will need to be completed by pupils and teachers. A computer room will need to be booked for pupils.	Jan 20

	**and Pupil Surveys (Time 3)**		
	**2. Complete Sustainability Survey**	Those who have delivered the intervention will complete a survey on implementation and embedding the interventions in schools.	Jan-Feb 20
	**3. Case Study School visits**	Selected schools will take part in interviews, focus groups and observations with a small number of staff and pupils. Schools will be made aware of whether or not this is required in November 19.	Jan-Feb 20
**Payment**	**Schools submit invoice**	Schools completing all requirements are invited to invoice following completion of Final Follow-Up Data Collection. Schools will also receive a report on their pupils' wellbeing, a participation certificate and a letter of thanks from DfE.	March 20

File S2. Example Newsletter

INSPIRE
Education
for Wellbeing

Phase 6 - Final Data Collection

22 days remaining

Thank you to the schools that have already started the pupil surveys! All of the surveys for *Phase 6: Final Follow-Up Data Collection* are now live:

Survey name	Date survey live	To be completed by	Survey link sent to	Survey deadline
Pupil survey	7 January 2020	All participating pupils	Key Contact (along with pupil passwords)	14 February 2020
Staff survey	13 January 2020	All staff named on the Pupil and Staff List	Individual staff	14 February 2020
Current Mental Health Provision Survey	13 January 2020	Key Contact	Key Contact	14 February 2020

Pupil Surveys

The **key contact** at your school should have all the necessary information to complete the pupil surveys - this was sent by our data team in Manchester.

If you have any issues accessing the online link or the file containing pupil passwords, please contact [Research Team Email Address]. You can also give our data manager a call: [Data Manager Phone Number]

Key things to note are:

- **Computer rooms should be booked** - all pupils will need access to a computer/tablet.
- All of the pupils who undertook the surveys last academic year will need to complete the survey again.
- Pupils that have joined the school since the last round of data collection will *not* be required to do the survey.
- Please **open the Pupil List file in advance** of the scheduled lesson and test the survey link. The **password** has been sent to the **Second Contact**.
- The **deadline** for pupils completing the survey is **Friday 14 February**.

Staff surveys

PLEASE NOTE: staff survey is only for those named in the Pupil and Staff List.

- The **deadline** for this survey is also **Friday 14 February**.
- Staff have **been emailed a link and an individual password**.
- Please ask colleagues to **check their spam/junk folders** if they have not received this.

The survey should take no more than **5 minutes** to complete.

Current mental health provision survey

The **key contact** at your school should have received a link to this survey about the mental health and wellbeing provision in your school. A version of this survey was also completed at the very beginning of the project (nearly two years ago!) and it is important that we capture any changes to provision in your school.

The **deadline** for this survey is also **Friday 14 February**.

Once this final step is completed, your school will be able to:

- **Invoice us**
- **Receive a tailored report about your pupils' mental health and wellbeing**
- **Receive a letter for thanks from DfE and a participation certificate**
